# 

**Published:** 1887-06

**Authors:** 


### CONTENTS.

### COMMUNICATIONS.

The Use and Abuse of the Teeth........ 265
Secondary or New Formation of Teeth ........ 268

### PROCEEDINGS.

Michigan State Dental Society........ 272

### SELECTIONS.

The Irradiation of Motor Impulses........ 282
Tumor of Parotid Gland........ 286
Notes on Dental Hygiene........ 289
Parotitis—Mumps........ 295
Parotid Tumor........ 297
Mania for Novelties in Medicine........ 298
Hospital Reports........ 300
Physiological Action of Nitrous Oxide Gas........ 302
Papayotin in Fissures of the Tongue........ 303
Should Physicians Extract Teeth?........ 304
Medical Cases in Court........ 307
Antiseptic Collodion........ 308
Calendula as a Styptic, Etc........ 309

CORRESPONDENCE........ 309

SALMAGUNDI........ 311

### EDITORIAL.

American Dental Association........ 314
A New Record Diagram........ 315
International Medical Congress........ 316

